# Chemical Composition, Enantiomeric Distribution and Biological Activity of Essential Oil from *Morella pubescens* (Humb. & Bonpl. ex Willd.) Wilbur

**DOI:** 10.3390/molecules28072910

**Published:** 2023-03-24

**Authors:** Eduardo Valarezo, Carlos Correa-Jaramillo, Paola Astudillo-Dávila, Julio Garzón-Yaguache, Luis Cartuche, Miguel Angel Meneses, Vladimir Morocho

**Affiliations:** Departamento de Química, Universidad Técnica Particular de Loja, Loja 110150, Ecuador

**Keywords:** biological activity, chemical composition, enantiomeric distribution, essential oil, *Morella pubescens*

## Abstract

The species *Morella pubescens*, commonly known as wax laurel, is a tree belonging to the Myricaceae family that can be found from Costa Rica to Bolivia. In this study, the chemical composition, enantiomeric distribution, and biological activity of essential oil isolated from the leaves of this species was determined. Hydrodistillation was used to isolate the essential oil (EO). Gas chromatography coupled with mass spectrometry was used to determine the qualitative composition, gas chromatography equipped with a flame ionization detector was used to determine quantitative composition, and gas chromatography on an enantioselective column was used to determine enantiomeric distribution. The broth microdilution method was employed to assess the antibacterial capacity of the essential oil against seven opportunistic microorganisms, including three Gram-positive cocci bacteria, a Gram-positive bacilli bacterium and three Gram-negative bacilli bacteria. 2,2′-azinobis-3-ethylbenzothiazoline-6-sulfonic acid radical cation and 2,2-diphenyl-1-picrylhydryl free radical were used as reagents to determine the antioxidant activity of essential oil. The spectrophotometric method was used to analyze the acetylcholinesterase inhibitory effect of the essential oil. The extraction method afforded a low yield of around 0.076 ± 0.008% (*v*/*w*). Fifty-eight chemical compounds, which represent 97.9% of the total composition, were identified in the essential oil. Sesquiterpene hydrocarbons were the most representative group with 24 compounds (67.8%). The principal constituents were (*E*)-caryophyllene (27.5 ± 1.3%), limonene (11.8 ± 0.6%), *δ*-selinene (9.1 ± 0.2%), *β*-selinene (8.0 ± 0.2%), selina-3,7(11)-diene (5.3 ± 0.2%) and germacrene B (5.0 ± 0.5%). Three pairs of enantiomers were identified in the essential oil of *Morella pubescens*. Essential oil presented strong activity against the bacterium *Enterococcus faecium (ATCC 27270)* with an MIC of 250 μg/mL. The antioxidant activity of essential oil was very strong in the ABTS method with an SC_50_ of 46.4 ± 1.0 µg/mL and was strong in the DPPH method with an SC_50_ of 237.1 ± 1.8 µg/mL. Additionally, the essential oil reported strong anticholinesterase activity with an IC_50_ of 133.5 ± 1.06 µg/mL.

## 1. Introduction

Medicinal plants are the first line of defense in the remediation of diseases, and are widely used globally, especially in developing countries where it is the only available therapeutic remedy [1]. The World Health Organization (WHO) defines traditional medicine as the sum total of the knowledge, skill, and practices based on the theories, beliefs, and experiences that are indigenous to different cultures, whether explicable or not, used in the maintenance of health as well as in the prevention, diagnosis, improvement, or treatment of physical and mental illness. It includes diverse health practices that incorporate plant, animal, and/or mineral-based medicines to maintain well-being, as well as to treat, diagnose and prevent disease. The study of phytochemicals in medicinal plants is of continuous interest for the validation of its traditional uses; both the study of their chemical compositions and bioactivities allow establishing a relation with their medical and pharmaceutical effects [2]. Many medicinal plants are also aromatic, so they also contain volatile secondary metabolites or essential oils. Medicinal and aromatic plant species are widely distributed throughout the world and form part of all existing botanical families [3].

Myricaceae Rich. ex Kunth is a small family of vascular plants containing five genera and approximately 60 species. Among the genera of this family are *Canacomyrica*, *Cerothamnus*, *Comptonia*, *Morella*, and *Myrica* [4]. The species of this family are shrubs or small trees, usually aromatic and resinous, that have simple, alternate, or pinnatifid leaves with generally unisexual flowers [5]. These species are found in temperate zones, subtropical zones, and in the tropics, mainly in mountainous areas [6]. In Ecuador, there are only two naturally occurring species of this family. These native species belong to the genus *Myrica* (*Myrica parvifolia* Benth. and *Myrica pubescens* Humb. & Bonpl. ex Willd.) [7]. The *Morella* Lour is a genus of the Myricaceae family, to which belong nine species located around the world, mainly in tropical and subtropical regions and in mountainous areas and moors of America, Europe, Africa, and Asia [8], differentiating itself from the rest of its genus for being the only monoecious in its group. This genus includes shrubs and trees with very attractive foliage grown as ornamentals. Some individuals contain astringent substances in the bark of their roots, which are used to induce vomiting. The fruits of certain species have been used as foodstuffs and in medicine for various purposes, but above all, they have been highly appreciated for the extraction of aromatic waxes [6]. One of the best-known species of this genus is *Morella cerifera* (L.) Small. *Morella cerifera* (L.) Small, which in addition to the production of wax, is commonly used as an astringent, diaphoretic, circulatory stimulant, and is used as a remedy for convulsions, colds, digestive system disorders, diarrhea, dysentery, leukorrhea, mucous colitis, measles, scarlet fever, nasal catarrh, jaundice, stomatitis, sore throat, irritable bowel syndrome, and ulcerative colitis [8]. Because its bark contains large amounts of tannins, they were extracted commercially. Most members of the family have nitrogen-fixing microorganisms in their roots [6].

*Morella pubescens* (Humb. & Bonpl. ex Willd.) Wilbur (class: Equisetopsida C. Agardh; subclass: Magnoliidae Novák ex Takht.; superorder: Rosanae Takht.; order: Fagales Engl.; family: Myricaceae Rich. ex Kunth; genus: Morella Lour.) is a species native to Costa Rica, Colombia, Peru, and Bolivia. In Ecuador, this species has been introduced and cultivated. This species is distributed between 1000–3500 m above sea level, especially in the Andean region and in the Altiplano. The species *M. pubesncens* is known by the common names “wax laurel”, “cebo”, “cebillo”, “laurel”, “laurel de cera”, “sittu”, “laurel de cerro”, “laurel grande”, and “cardi laurel” [9,10]. This species appears as a perennial tree or shrub that grows up to 4 m in height. The trunk is light brown, rounded, short, and crooked. It has a wide, irregular crown with dense foliage, abundant with lanceolate leaves of a yellowish olive-green color and serrated margin, which exhale a pleasant odor when squeezed. The flowers are minute and appear in catkins. The fruits are fleshy and covered with scales that contain a whitish wax. The leaves of *M. pubescens* are edible. The stem is used in the manufacture of plows, the elaboration of handicrafts and images, in the construction of houses, and also to make charcoal [11]. The infusion of the leaves is taken to combat fatigue, to regulate menstruation, to combat nervous weakness, to treat the initial phase of deafness, as well as for childbirth and postpartum conditions. The young leaves are consumed to treat muscle pain caused by prolonged work and the infusion is used to treat rheumatism in the affected areas [10].

Even though the *Morella* species are known to possess essential oils, the bibliography on this subject is scarce; up until now, only the study of the essential oil of three species of this genus has been reported [12,13,14,15]. Arango, et al. in 2009 [15] and Sandoval, et al. in 2010 [12] reported the existence of essential oil in the *M. pubescens* species collected in Colombia; however, there is no literature to date on the enantiomeric composition and biological activities of this essential oil. This fact has motivated us to carry out the present study with the aim of determining the chemical composition and enantiomeric distribution of the essential oil isolated from the leaves of *Morella pubescens*, as well as its antibacterial, antioxidant and anticholinesterase activities. In this way, we can contribute information on the aromatic and medicinal flora of Ecuador.

## 2. Results

### 2.1. Essential Oil Obtained

A total of 15,000 g (three distillations of 5000 g) of fresh (with a moisture of 72 ± 4% *w*/*w*) *M. pubescens* leaves were hydrodistilled in a Clevenger-type apparatus to isolate its essential oil (EO). The amount of EO obtained was 11.4 mL, which represents a yield of 0.076 ± 0.008% (*v*/*w*), or 0.76 ± 0.08 mL/Kg.

### 2.2. Physical Properties of Essential Oil

The EO from *M. pubescens* leaves presented as an unctuous liquid with a strong odor characteristic of this species. Table 1 shows the mean values and standard deviations (SD) of the physical properties of essential oil. In general, the essential oil of *M. pubescens* was a yellow liquid less dense than water.

### 2.3. Chemical Composition of Essential Oil

The qualitative identification of the *M. pubescens* compounds was carried out by means of gas chromatography coupled with mass spectrometry (GC-MS), and the quantification of their relative abundances was made by means of gas chromatography equipped with the flame ionization detector (GC-FID). The compound number (CN) assigned according to their elution order, retention time (RT), calculated retention indices (RIC), references retention indices (RIR), relative abundance (%), chemical formula (CF), and monoisotopic mass for each compound are shown in Table 2. A total of 58 chemical compounds were identified in the EO of leaves from *M. pubescens*, which represent 97.94% of the total composition. The compounds were classified into five groups: monoterpene hydrocarbons (MH), oxygenated monoterpenes (OM), sesquiterpene hydrocarbons (SH), oxygenated sesquiterpene (OS), and other compounds (OC). Sesquiterpene hydrocarbons was the most represented group, with twenty-four compounds at 67.8%, followed by monoterpene hydrocarbons with 11 compounds, reaching 19.7%. The oxygenated monoterpenes were the least represented group in abundance, and the presence of diterpenes was not determined. The principal constituents (>5%) are found to be sesquiterpene hydrocarbons (*E*)-caryophyllene (CN: 27, CF: C_15_H_24_, MM: 204.19 Da) at 27.5 ± 1.3%, stereoisomers δ- and β- of selinene (CF: C_15_H_24_, MM: 204.19 Da) at 9.1 ± 0.2% and 8.0 ± 0.2%, respectively, selina-3,7(11)-diene at 5.3 ± 0.2%, germacrene B at 5.0 ± 0.5%, and MH limonene (mixture of (+) and (−) enantiomers (CN: 10, CF: C_10_H_16_, MM: 136.13 Da) with 11.8 ± 0.6%.

### 2.4. Enantiomeric Analysis

Using a column with the enantioselective stationary phase, it was possible to separate three pairs of enantiomers from the EO of *M. pubescens* leaves. Table 3 shows the retention time (RT), enantiomers, retention indices (RI), enantiomeric distribution (ED), and enantiomeric excess (e.e.), for each pair of compounds. The (−)-α-pinene and (+)-limonene were found to be practically pure with 94.8% and 91.3% of enantiomeric excess, respectively.

### 2.5. Antimicrobial Activity

The microdilution broth method was used to determine the antibacterial activities of EO from leaves from *M. pubescens*. Table 4 shows the tested microorganisms and minimum inhibitory concentration (MIC) values of both the EO and positive control. The values of the negative control are also shown. Ampicillin was used as a positive control for *Enterococcus faecalis*, *Enterococcus faecium*, and *Staphylococcus aureus,* and ciprofloxacin was used as a positive control for *Listeria monocytogenes*, *Escherichia coli*, *Pseudomonas aeruginosa*, and *Salmonella enterica.* The *M. pubescens* EO reported MIC values of 250 µg/mL against *Enterococcus faecium,* 2000 µg/mL against *Staphylococcus aureus*, and 4000 µg/mL against *Listeria monocytogenes*.

### 2.6. Antioxidant Activity

The antioxidant activity of essential oil from *M. pubescens* was determined using the methods DPPH and ABTS. Table 5 shows the scavenging capacity (SC_50_) in µg/mL of both the essential oil and the positive control. The maximum evaluated concentration was 1000 µg/mL. *M. pubescens* EO presented a SC_50_ of 46.37 µg/mL with method ABTS, a value close to that of the positive control.

### 2.7. Anticholinesterase Activity

**Figure 1:**
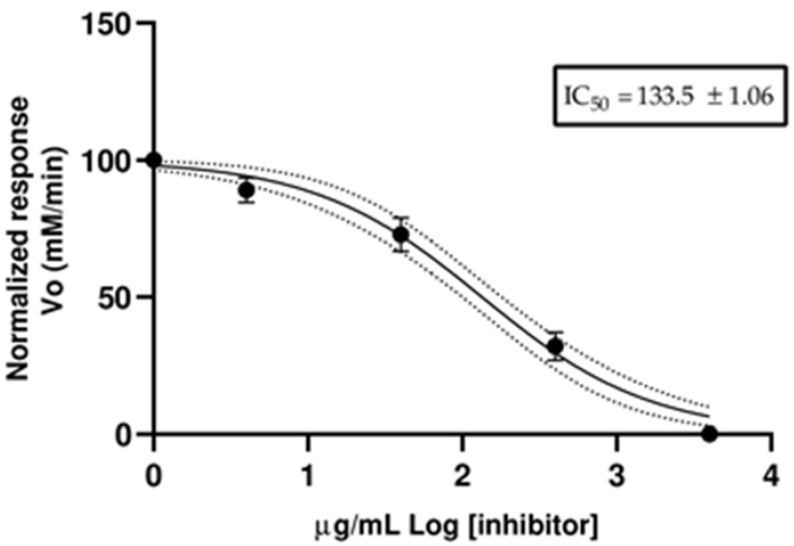
Anticholinesterase activity of essential oil from *Morella pubescens*.

The anticholinesterase activity was determined using a spectrophotometric method. Figure 1 shows the Log of the concentration of EO, and the normalized response rate of the reaction of acetylcholinesterase. The results were reported as a half-maximal inhibitory concentration (IC_50_) value. The *M. pubescens* EO reported an IC_50_ value of 133.5 ± 1.1 µg/mL. The positive control (donepezil) exhibited an IC_50_ value of 12.4 ± 1.4 µg/mL.

## 3. Discussion

The extraction yield of EO was 0.076 ± 0.008% (*v*/*w*), which could be considered a very low yield [16]. No reports about this value have been published in scientific articles for *M. pubescens*, but a preliminary work [17] reported a value of 0.24% for samples collected in Perú. The extraction yield is recognized to be dependent on the extraction process. Arango et al. [15] reported in 2009, regarding the hydrodistillation of *M. pubescens*, that the interaction between particle size and extraction time has an influence on the concentration of chemical components of the essential oil. Clearly, this is related to the extraction yield, but they did not report the value. For other species of this genus, Dolveni et al. published in 2016 an extraction yield between 0.3% to 0.5% for the essential oil of *Morella parvifolia* (Benth.) Parra-Os. [14]. The physical properties and the chemical composition could both be considered a characteristic of purity of the EO, as in this case for *M. pubescens*, the variations of refractive index are associated with changes in the chemical composition—as mentioned by Delgado Ospina, et al., this could allow its use as a quality parameter [18].

The chemical composition of EO *M. pubescens* has been reported previously by Quijano Celis and Pino [19] in 2007. They identified 121 compounds representing 95% of total compounds, where the main components were 1,8-cineol (20.0%), linalool (16.4%), and α-terpinyl acetate (11.1%) for the EO of samples collected in Cuba. In 2009, Arango et al. [15] reported the composition of EO for samples collected in Colombia; the main compounds were *trans*-caryophyllene (21.3%), α-selinene (10.7%), β-selinene (10.0%), and caryophyllene oxide (4.8%). Furthermore, in 2010, Sandoval et al. [12] identified 55 compounds that represented 94% of the EO. The main compounds were reported as germacrene-B (30.9%), selina-3,7(11)-diene (17.4%), δ-cadinene (14.7%), valencene (6.4%) and γ-elemene (4.8%). In this article, the main compounds were (*E*)-caryophyllene (27.5%), limonene (11.8%), δ-selinene (9.07%), β-selinene (8.0%), selina-3,7(11)-diene (5.3%) and germacrene B (5.0%). The main compounds were relatively similar to those reported by Arango et al. [15], for both the most abundant was (*E*)-caryophyllene. In this research, from the fifty-eight compounds identified in the EO of *M. pubescens*, 67.8% were grouped as sesquiterpene hydrocarbons. A similar result was observed by Sandoval et al. in 2010 [12], who also mentioned the similarities to the EO obtained from the fruit. Nevertheless, for the same species, the similarities or differences in the chemical composition of EO should be considered with detail; there are several factors that modified the presence and the quantity of chemical compounds, such as extraction procedure, the age of plant, maturity, location, weather, florescence, among other factors regarding farming practices.

Regarding the chemical composition in another *Morella* species, Dolveni et al. in 2016 presented the main components of EO *M. parvifolia* as α-bisabolol (50.6–58.9%) and α-pinene (12.9–16.8%). These values are different for the present study (α-pinene 4.0%), and the reported by Quijano et al. (2007) (α-pinene 2.9%) [14].

The enantiomeric analysis allows us to consider the potential applications of EO in pharmaceutical or food products [20] if bioactive chiral compounds are present in the complex mixture of an essential oil. For the EO of *M. pubescenes*, the occurrence of three pairs of enantiomers is reported in Section 2, and this is the first report of enantioselectivity GC-MS analysis.

The previous studies of *M. pubescens* did not perform analysis of bioactivity of the EO, and this study is the first to report on antibacterial, antioxidant, and anticholinesterase analyses. According to the criteria published by Van Vuuren and Holl in 2017 [21] about a scale for the MIC values of plant extracts and essential oils, the EO of *M. pubescens* (MIC 250 µg/mL) shows strong activity (MIC 101 to 500 µg/mL) against *Enterococcus faecium* (ATCC 27270), but it was inactive (MIC > 1001 µg/mL) against the other microorganisms tested (Table 4). As a comparation, let us present the antibacterial activity of other *Morella* species. Dolveni et al. in 2016 reported no antibacterial activity regarding the EO of *M. parvifolia* [14], in the same way Setzer et al. in 2006 reported that the antibacterial activity against the EO of *Morella cerifera* (L.) Small (syn. *Myrica cerifera*) was strong (MIC 312 µg/mL) against *Escherichia coli*, moderate (MIC 625 µg/mL) against *Bacillus cereus*, *Staphylococcus aureus*, *Pseudomonas aeruginosa*, *Candida albicans*, and *Aspergillus niger*, and that the most abundant components were 1,8-cineole (30.7%) and α-terpineol (14.2%) [22]. Meniso et al. in 2019 studied the EO of *Morella salicifolia* (Hochst. ex A. Rich.) Verdc. and Polhill, and reported antibacterial activity against *S. aureus*, *Streptococcus agalactiae*, *E. coli*, and *Shigella flexneri*, where the main components were hexadecanoic acid methyl ester (29.4%), (*Z*)-9-octadecenoic acid methyl ester (28.6%), and methyl tetradecanoate (10.7%) [23]. It is difficult to associate the antibacterial activity to the major components in an essential oil even though the individual components have shown strong antibacterial activity [24,25]. Rather, the additive or synergistic effects become an antagonistic one, causing a decrease or loss of activity. Lis-Balcnin et al. reported that 18 out of 25 different bacteria were more affected by the (−) enantiomer of α-pinene, while the (+) isomer affected more, about 19 out of 20 *L. monocytogenes* strains [26]. The different bioactivities could be referring to the different enantiomeric ratio of chiral compounds, and this was observed by Van Vuuren and Viljoen in 2007, who evaluated the antibacterial activity of both enantiomers (+) and (−) of limonene, and the combination with 1,8-cineole [27].

Regarding the antioxidant activity, the EO of *M. pubescenes* showed strong activity by the ABTS assay while the value was weak for the DPPH assay. In the literature, essential oils have shown strong, moderate, low, or no antioxidant activity. Anthony et al. in 2012 [28] reported that mono- and sesquiterpenes have less antioxidant activity after phenol compounds. The difference in composition explains the antagonist or synergistic activity, which was observed by Chandra et al. in 2017 [29]. Dahham et al. reported a strong antioxidant activity for β-caryophyllene with 1.3 ± 0.1 µM [25]. The antioxidant activity of limonene has been demonstrated to effectively attenuate oxidative stress in diabetic rats [30].

The anticholinesterase activity of the EO *M. pubescens* has not been published before. The EO of *M. pubescens* present a strong AChE inhibitory effect with an IC_50_ of 133.5 ± 1.1 µg/mL, and this result is less than the reported for other EO, such as *Annona cherimola* Mill. (IC_50_ 41.5 µg/mL) [31], *Piper carpunya* Ruiz & Pav. (IC_50_ 36.4 µg/mL) [32], and *Diplostephium juniperinum* Cuatrec. (IC_50_ 67.2 µg/mL) [33]. The results of AChE are relevant in researching treatments for Alzheimer disease; Benny and Tomas in 2019 reported the neuroprotective effect of EO and their pure compounds [34].

## 4. Materials and Methods

### 4.1. Materials

Helium was purchased from INDURA (Quito, Ecuador). Mueller Himton broths, Mueller Hinton II broths, and fluid thioglycollate medium were purchased from DIPCO (Quito, Ecuador). The standard aliphatic hydrocarbons were purchased from ChemService (West Chester, PA, USA). Acetylcholinesterase (AChE), acetylthiocholine (AcSCh), dichloromethane (DMC), dimethyl sulfoxide (DMSO), methanol (MeOH), 2,2-diphenyl-1-picrylhydryl (DPPH), 2,2′-azinobis-3-ethylbenzothiazoline-6-sulfonic acid (ABTS), 5,5′-dithiobis (2-nitrobenzoic acid) (DTNB), butylated hydroxytoluene (BHT), donepezil, magnesium chloride hexahydrate, phosphate buffered saline (PBS), sodium sulfate anhydrous, trolox, and tris hydrochloride (Tris-HCl) were purchased from Sigma-Aldrich (St. Louis, MO, USA). All chemicals were of analytical grade and used without further purification.

### 4.2. Plant Material

The leaves of *M. pubescens* were collected in the surroundings of the Guayllabamba parish, Quito canton, Pichincha province. The collection was carried out in a valley that is located at 0°04′43″ south longitude and 78°20′59″ west latitude, and at an altitude of 2171 m above sea level. After being collected, the plant material was stored and transferred in airtight plastic containers. The environmental conditions in the collection and transfer were a pressure of 79 KPa and a temperature of 18–20 °C.

### 4.3. Essential Oil Isolation

A Clevenger-type apparatus was used for the isolation of essential oil. The extraction of the oil was carried out by hydrodistillation according to the procedures previously described by Valarezo et al. [31], for which an 80 L distiller with approximately 18 L of water was used. The process was maintained for 3 h, counting from the fall of the first drop of distillate. The condensed essential oil was separated from the water by decantation, then it was dried using anhydrous sodium sulfate and stored at 4 °C in amber sealed vials until being used in analysis.

### 4.4. Identification and Quantification of Essential Oil Compounds

The analysis of chemical composition was carried out in a gas chromatograph (GC) (model 6890N series, Agilent Technologies, Santa Clara, CA, USA). For qualitative analysis, the GC was coupled to a quadrupole mass spectrometer (MS) (model Agilent series 5973 inert, Agilent Technologies, Santa Clara, CA, USA), and for quantitative analysis, and the GC was equipped with a flame ionization detector (FID). In both cases, a nonpolar chromatographic column (Agilent J&W DB-5ms Ultra Inert GC column, Agilent Technologies, Santa Clara, CA, USA) with stationary phase 5%-phenyl-methylpolyxilosane, 30 m of length, 0.25 mm of internal diameter, and 0.25 µm of stationary phase thickness was used. The GC was equipped with a split/splitless autosampler (model 7683, Agilent Technologies, Santa Clara, CA, USA). The supply of hydrogen for the FID was carried out using a gas generator (model 9150, Packard, Detroit, MI, USA). The EO sample was prepared at 1% (*v*/*v*), putting 10 μL of EO and 990 μL of dichloromethane in an amber vial. For the qualitative and quantitative analyses, 1 μL of sample was injected in split mode with a partition ratio of 40:1, at a temperature of 220 °C, and at a pressure of 11 psi. In both cases, the chromatographic run began maintaining the initial temperature of 50 °C for 3 min, then the temperature was increased 3 °C/min until reaching a final temperature of 230 °C, which was maintained for 3 min. For GC-MS, a constant flow of helium was maintained at a rate of 0.9 mL/min and a velocity of 23 cm/s, and for GC-FID, the flow was 1.0 mL/min and the speed was 40 cm/s. Equation (1) [35] was used to determine the retention index (RI) of each compound. For the identification of the compounds, the IR and the mass spectra were compared with those in the bibliography [36,37].

(1) $$\mathrm{RI}=100C+100\frac{\left(\mathrm{RTx}-\mathrm{RTn}\right)}{\left(\mathrm{RTN}-\mathrm{RTn}\right)}$$

where C is the carbon number of aliphatic hydrocarbons (C_9_ to C_25_) that elutes after before of the compound of interest, RTx is the retention time of the compound of interest, RTn is the retention time of aliphatic hydrocarbons that elutes before the compound of interest, and RTN is the retention time of hydrocarbons that elutes after the compound of interest.

### 4.5. Enantioselective Analysis

For enantiomeric analysis, a gas chromatography (Trace 1310, Thermo Fisher Scientific, Waltham, MA, USA) coupled to a mass spectrometer (quadrupole) (ISQ 7000, Thermo Fisher Scientific, Waltham, MA, USA) was used. Analyses were performed on an enantioselective GC column (MEGA-DEX DMT-Beta, Mega, Legnano, MI, Italy) with 30 m of length, 0.25 m of internal diameter, and 0.25 μm of thick stationary phase (2.3 -diethyl-6-tert-butyldimethylsilyl-β-cyclodextrin). Sample preparation, amount injected, injection temperature, and partition radius were similar to those described for GC-MS. The carrier gas used was helium with a flow of 1.0 mL/min and a speed of 40 cm/s. The chromatographic run began maintaining the oven at 60 °C for 5 min, then the temperature was increased with a ramp of 2 °C/min up to 230 °C. finally, this temperature was maintained for 5 min. The calculation of the enantiomeric excess and elution order was carried out according to the procedures previously described by Morocho et al. [38].

### 4.6. Antimicrobial Activity

The antibacterial activity of the essential oil was tested against seven opportunistic and nosocomial bacteria that are commonly found in hospitals, or which act as saprofitic organisms and can lead to a variety of infections in vital organs or systems such as the lungs, heart, urinary tract, gastrointestinal tract, skin, etc. Three Gram-positive cocci bacteria: *Enterococcus faecalis* (ATCC 19433), *Enterococcus faecium* (ATCC 27270), and *Staphylococcus aureus* (ATCC 25923); a Gram-positive bacilli bacterium: *Listeria monocytogenes* ATCC 19115; and three Gram-negative bacilli bacteria: *Escherichia coli* O157:H7 (ATCC 43888), *Pseudomonas aeruginosa* (ATCC 10145), and *Salmonella enterica* subs enterica serovar *Thypimurium* WDCM 00031, derived from (ATCC 14028), were included in the assay. The broth microdilution method was used to determine this activity, and the procedures were performed as previously described by Valarezo et al. [39]. The maximum evaluated concentration was 4000 µg/mL. Ampicillin and ciprofloxacin were used as a positive control, and DMSO was used as a negative control.

### 4.7. Evaluation of Antioxidant Capacity

The DPPH and ABTS methods were used to determine free radical scavenging activity of EO from *M. pubescens.* The DPPH method is based on the scavenging capacity of the essential oil against the radical 2,2-diphenyl-1-picrylhydrazyl (DPPH^•^), and the ABTS scavenging capacity was determined against the radical ion 2,2’-azino-bis (3-ethylbenzothiazoline-6-sulfonic acid) (ABTS^•+^). The antioxidant capacity of EO was determined according to the procedure described by Salinas et al. [33], using a UV spectrophotometer (Genesys 10S UV-Vis Spectrophotometer, Thermo Fisher Scientific, Waltham, MA, USA). In the DPPH method, 2,2-diphenyl-1-picrylhydrazyl radical (DPPH^•^) was produced from the reagent 2,2-diphenyl-1-picrylhydrazyl (DPPH), and the absorbance of the samples was measured at a wavelength of 515 nm. Instead, in the ABTS method, 2,2’-azinobis(3-ethylbenzothiazoline-6-sulfonic acid) radical cation (ABTS^•+^) was produced from the reagent 2,2’-azinobis (3-ethylbenzothiazoline-6-sulfonic acid) (ABTS), and the measurement of the absorbance of the samples was carried out at a wavelength of 734 nm. SC_50_, which is the concentration value necessary for the EO to have half-radical scavenging capacity, was used to express the antioxidant activity. Trolox and methanol were used as a positive and negative control, respectively.

### 4.8. Anticholinesterase Activity

The spectrophotometric method was used to determine the acetylcholinesterase inhibitory effect of the EO of leaves from *M. pubescens*. The procedures were performed according to what was previously described by Valarezo et al. [32]. Measurements were made in a microplate spectrophotometer (EPOCH 2, BioTek, Winooski, VT, USA) at a wavelength of 405 nm. The IC50 was used to express the anticholinesterase activity. IC50 is the concentration of EO required for 50% inhibition. Methanol and donepezil hydrochloride were used as a negative and positive control, respectively.

### 4.9. Statistical Analysis

All procedures were performed in triplicate, except for the identification of essential oil compounds, the enantioselective analysis, and identification of antimicrobial activity, which were performed nine times. The data were collected in a Microsoft Excel spreadsheet. The statistical software Minitab 17 (Version 17.1.0., Minitab LLC., State College, PA, USA) was used to calculate the measures of central tendency and standard deviation.

## 5. Conclusions

The enantiomeric distribution, antimicrobial activity, antioxidant capacity, and anticholinesterase activity of essential oil from leaves of *Morella pubescens* was determined for the first time. Fifty-eight chemical compounds and three pairs of enantiomers were identified in the essential oil. The main compound was (*E*)-caryophyllene. Essential oil presented strong activity against Enterococcus faecium, and very strong antioxidant activity. With this research, new information is provided on the species of aromatic plants of Ecuador, thus contributing to the knowledge of Ecuadorian biodiversity. The biological activities displayed by the essential oil of leaves from *Morella pubescens* make this essential oil novel for the cosmetic, food, and pharmaceutical industries. For future studies, based on the good results of in vitro activity, it is proposed that in vivo studies be carried out—for example regarding anti-inflammatory activity in mice.

## Figures and Tables

**Table 1:** Physical properties of the essential oil of *Morella pubescens*.

	*Morella pubescens* EO	
	Mean	SD
Density, ρ (g/cm^3^)	0.8978	0.0039
Refractive index, *n*^20^	1.4976	0.0006
Specific rotation, [α] (°)	+1.04	0.05
Subjective color	Yellow	
RGB color values	R:255, G:255, B:141	
CMYK color values	C:0, M:0, Y:0.45, K:0	

**Table 2:** Chemical composition of essential oil from the leaves of *Morella pubescens*.

CN	RT	Compound	RIC	RIR	% ^a^	SD	Type	CF	MM (Da)
1	7.91	*α*-Thujene	924	924	0.1	0.0	MH	C_10_H_16_	136.13
2	8.18	*α*-Pinene	933	932	4.0	0.2	MH	C_10_H_16_	136.13
3	8.83	Camphene	946	946	0.1	0.0	MH	C_10_H_16_	136.13
4	9.80	Sabinene	969	969	0.1	0.0	MH	C_10_H_16_	136.13
5	9.98	*β*-Pinene	974	974	0.3	0.0	MH	C_10_H_16_	136.13
6	10.56	Myrcene	987	988	0.4	0.0	MH	C_10_H_16_	136.13
7	11.23	n-Octanal	1000	998	0.1	0.0	OC	C_8_H_16_O	128.12
8	11.73	*α*-Terpinene	1015	1014	0.1	0.0	MH	C_10_H_16_	136.13
9	12.09	ρ-Cymene	1022	1020	1.2	0.1	MH	C_10_H_14_	134.11
10	12.30	Limonene	1026	1024	11.8	0.6	MH	C_10_H_16_	136.13
11	12.47	1,8-Cineole	1029	1026	0.3	0.0	OM	C_10_H_18_O	154.14
12	13.63	*γ*-Terpinene	1056	1054	1.5	0.1	MH	C_10_H_16_	136.13
13	14.89	Terpinolene	1083	1086	0.1	0.0	MH	C_10_H_16_	136.13
14	15.72	Linalool	1099	1095	0.1	0.0	OM	C_10_H_18_O	154.14
15	20.68	n-Decanal	1205	1201	tr	-	OM	C_10_H_20_O	156.15
16	20.93	Octanol acetate	1212	1211	tr	-	OM	C_10_H_20_O_2_	172.15
17	22.14	Neral	1239	1235	0.1	0.0	OM	C_10_H_16_O_2_	152.12
18	23.50	Geranial	1268	1264	0.3	0.0	OM	C_10_H_16_O_2_	152.12
19	24.24	(*E*)-Anethole	1285	1282	0.4	0.0	OM	C_10_H_12_O	137.00
20	27.03	*α*-Cubebene	1344	1348	tr	-	SH	C_15_H_24_	204.19
21	27.22	*α*-Terpinyl acetate	1348	1346	tr	-	OC	C_12_H_20_O_2_	196.15
22	28.34	α-Copaene	1372	1374	0.1	0.0	SH	C_15_H_24_	204.19
23	28.95	(*Z*)-*β*-Damascone	1385	1386	0.1	0.0	OC	C_13_H_20_O	208.15
24	29.09	*β*-Elemene	1388	1389	1.3	0.0	SH	C_15_H_24_	204.19
25	29.19	Sativene	1390	1390	0.2	0.0	SH	C_15_H_24_	204.19
26	29.66	*β*-Longipinene	1400	1400	0.2	0.0	SH	C_15_H_24_	204.19
27	30.40	(*E*)-Caryophyllene	1416	1417	27.5	1.3	SH	C_15_H_24_	204.19
28	30.87	(*E*)-*α*-Ionone	1426	1428	4.2	0.2	OC	C_13_H_20_O	208.15
29	31.43	6,9-Guaiadiene	1438	1442	0.1	0.0	SH	C_15_H_24_	204.19
30	32.00	*α*-Humulene	1450	1452	0.7	0.0	SH	C_15_H_24_	204.19
31	32.23	allo-Aromadendrene	1455	1458	0.1	0.0	SH	C_15_H_24_	204.19
32	32.70	4,5-di-*epi*-Aristolochene	1465	1471	0.2	0.0	SH	C_15_H_24_	204.19
33	32.88	*β*-Chamigrene	1469	1476	1.1	0.0	SH	C_15_H_24_	204.19
34	32.98	*trans*-Cadina-1(6),4-diene	1471	1475	0.3	0.0	SH	C_15_H_24_	204.19
35	33.35	ar-Curcumene	1479	1479	0.2	0.0	SH	C_15_H_24_	202.17
36	33.45	*γ*-Himachalene	1481	1481	0.2	0.0	SH	C_15_H_24_	204.19
37	33.59	*β*-Selinene	1484	1489	8.0	0.2	SH	C_15_H_24_	204.19
38	33.91	*δ*-Selinene	1491	1492	9.1	0.2	SH	C_15_H_24_	204.19
39	34.10	*α*-Muurolene	1495	1500	0.2	0.0	SH	C_15_H_24_	204.19
40	34.38	*γ*-Patchoulene	1501	1502	0.1	0.0	SH	C_15_H_24_	204.19
41	34.57	*β*-Bisabolene	1505	1505	0.2	0.0	SH	C_15_H_24_	204.19
42	34.76	*γ*-Cadinene	1509	1513	0.3	0.0	SH	C_15_H_24_	204.19
43	34.99	*δ*-Cadinene	1514	1520	2.9	0.1	SH	C_15_H_24_	204.19
44	35.65	Lilial	1528	1527	0.8	0.0	OC	C_14_H_20_O	204.15
45	35.83	Zonarene	1532	1528	4.7	0.2	SH	C_15_H_24_	204.19
46	36.07	Selina-3,7(11)-diene	1537	1545	5.3	0.2	SH	C_15_H_24_	204.19
47	36.35	Occidentalol	1543	1550	0.0	0.0	OS	C_15_H_24_O	220.18
48	36.82	Germacrene B	1553	1559	5.0	0.5	SH	C_15_H_24_	204.19
49	37.00	*epi*-Longipinanol	1557	1562	0.3	0.0	OS	C_15_H_26_O	222.20
50	37.94	Caryophyllene oxide	1577	1582	1.8	0.1	OS	C_15_H_24_O	220.18
51	40.19	2-*epi*-α-Cedren-3-one	1625	1626	0.3	0.0	OS	C_15_H_22_O	218.17
52	40.79	*epi*-*α*-Cadinol	1638	1638	0.2	0.0	OS	C_15_H_26_O	222.20
53	41.03	Selina-3,11-dien-6-α-ol	1643	1642	0.3	0.0	OS	C_15_H_24_O	220.18
54	41.54	*α*-Cadinol	1654	1652	0.7	0.0	OS	C_15_H_26_O	222.20
55	42.01	Intermedeol	1664	1665	0.2	0.0	OS	C_15_H_26_O	222.20
56	43.37	Eudesm-7(11)-en-4-ol	1693	1700	0.4	0.0	OS	C_15_H_26_O	222.20
57	43.98	(*E*)-Apritone	1706	1708	0.1	0.0	OS	C_15_H_24_O	220.18
58	44.12	14-hydroxy-*α*-Humulene	1709	1713	tr	-	OS	C_15_H_24_O	220.18
MH					19.7				
OM					1.2				
SH					67.8				
OS					4.1				
OC					5.2				
Total identified					97.9				

^a^: mean of nine replicates (three collections per three extractions); SD: standard deviation; Tr: traces.

**Table 3:** Chiral compounds present in the essential oil of the leaves from *Morella pubescens*.

RT	Enantiomers	RI	ED (%)	e.e. (%)
4.81	(+)-α-Pinene (1R,5R)	917	2.6	94.8
5.01	(−)-α-Pinene (1R,5R)	923	97.4	
9.56	(−)-Limonene (4S)	1037	4.3	91.3
9.71	(+)-Limonene (4R)	1040	95.7	
39.07	(+)-δ-cadinene (1S,8aR)	1539	17.6	64.8
39.17	(−)-δ-cadinene (1R,8aS)	1541	82.4	

**Table 4:** Antibacterial activity of essential oil from *Morella pubescens*.

Microorganism	Essential oil	Positive Control	Negative Control
	MIC (µg/mL)		
Gram-Positive Cocci			
*Enterococcus faecalis* (ATCC 19433)	>4000	0.78	+
*Enterococcus faecium* (ATCC 27270)	250	0.39	+
*Staphylococcus aureus* (ATCC 25923)	2000	0.39	+
Gram-positive bacilli			
*Listeria monocytogenes* ATCC 19115	4000	1.56	+
Gram-negative bacilli			
*Escherichia coli* O157:H7 (ATCC 43888)	>4000	1.56	+
*Pseudomonas aeruginosa* (ATCC 10145)	>4000	0.39	+
*Salmonella enterica* subs enterica serovar *Thypimurium* WDCM 00031, derived (ATCC 14028)	>4000	0.39	+

+: normal growth.

**Table 5:** Antioxidant activity of essential oil from *Morella pubescens*.

Sample	DPPH	ABTS
	SC_50_ (µg/mL) ± SD	
*Morella pubescens* essential oil	237.1 ± 1.8	46.4 ± 1.0
Trolox	30.0 ± 1.1	23.3 ± 1.1

## Data Availability

Data are available from the authors upon reasonable request.

## Sample Availability

Samples of essential oil are available from the authors.
